# Research on Global Optimization Algorithm of Integrated Energy and Thermal Management for Plug-In Hybrid Electric Vehicles

**DOI:** 10.3390/s23167149

**Published:** 2023-08-13

**Authors:** Junyu Jiang, Yuanbin Yu, Haitao Min, Weiyi Sun, Qiming Cao, Tengfei Huang, Deping Wang

**Affiliations:** 1 State Key Laboratory of Automotive Simulation and Control, Jilin University, Changchun 130022, China; jiangjy18@mails.jlu.edu.cn (J.J.); yyb@jlu.edu.cn (Y.Y.); swy_18@jlu.edu.cn (W.S.); caoqm20@mails.jlu.edu.cn (Q.C.); huangtf19@mails.jlu.edu.cn (T.H.); 2 China FAW Group Co., Ltd., Changchun 130013, China

**Keywords:** plug-in hybrid electric vehicle, global optimization, energy and thermal management, dynamic programming, energy-saving potential

## Abstract

Power distribution and battery thermal management are important technologies for improving the energy efficiency of plug-in hybrid electric vehicles (PHEVs). In response to the global optimization of integrated energy thermal management strategy (IETMS) for PHEVs, a dynamic programming algorithm based on adaptive grid optimization (AGO–DP) is proposed in this paper to improve optimization performance by reducing the optimization range of SOC and battery temperature, and adaptively adjusting the grid distribution of state variables according to the actual feasible region. The simulation results indicate that through AGO–DP optimization, the reduction ratio of the state feasible region is more than 30% under different driving conditions. Meanwhile, the algorithm can obtain better global optimal driving costs more rapidly and accurately than traditional dynamic programming algorithms (DP). The computation time is reduced by 33.29–84.67%, and the accuracy of the global optimal solution is improved by 0.94–16.85% compared to DP. The optimal control of the engine and air conditioning system is also more efficient and reasonable. Furthermore, AGO–DP is applied to explore IETMS energy-saving potential for PHEVs. It is found that the IETMS energy-saving potential range is 3.68–23.74% under various driving conditions, which increases the energy-saving potential by 0.55–3.26% compared to just doing the energy management.

## 1. Introduction

With the development of new energy technologies in the automotive industry, the plug-in hybrid electric vehicle (PHEV) has become one of the important approaches to reducing carbon emissions and the usage of fossil fuels and achieving the electrification of transport and sustainable development [1,2]. The PHEV has multiple power sources, including engine and battery, which can greatly alleviate the problem of ‘mileage anxiety’ in battery electric vehicles [3]. The PHEV has a larger battery capacity and a longer pure electric range than other hybrid electric vehicles. Meanwhile, it can directly supplement electricity from the power grid, with more energy-saving performance. Energy management among multiple power sources and battery thermal management are the crucial technologies for improving the performance of PHEVs.

The energy management strategies (EMS) of PHEV are used to reasonably and effectively distribute the output power between the engine and battery to meet the vehicle demand power [4,5], which can be divided into three categories: rule-based strategy, instantaneous optimization-based strategy, and global optimization-based strategy [6,7]. Rule-based strategy has the lowest computational complexity compared with other strategies, so it is widely applied in online vehicle control. Its control performance depends on logical rule formulation and parameter selection [8]. Although some studies have improved rules and introduced fuzzy logic for power distribution [9,10], it is still difficult to adapt to various real-world driving conditions. Optimization-based strategies require establishing an objective function and usually combine with vehicle speed prediction [11,12] and driving condition identification [13] to minimize the objective function within the optimization prediction horizon. Equivalent consumption minimization strategy [7,14] and model prediction control [5,15,16,17] are common instantaneous optimization-based strategies, which have better economic efficiency but much higher computational complexity than rule-based strategies. In particular, global optimization-based strategies consider the entire vehicle driving cycle as a predictive horizon for the optimization problem, which is committed to obtaining the global optimal solution. The global optimization-based strategy needs to know the actual driving condition in advance and take it as input to optimize the power distribution of PHEVs, such as dynamic programming (DP) [18], Pontriagin’s minimum principle [19], and the breadth-first search [20]. However, it inevitably increases the computational burden significantly, making it difficult to use for real-time vehicle control, although the global theoretical optimal solution obtained by DP through offline calculation is often used as a reference benchmark to evaluate the energy-saving potential of other online strategies. Otherwise, the optimal results of DP under different driving conditions are also applied to guide the development of online strategies for PHEVs. Liu et al. summarized the load-adaptive strategy rules by studying the relationship between demand power and battery output power in the DP optimal results [21]. The optimal SOC allocation method is proposed by Yu according to the minimum fuel consumption distribution under different types of driving conditions obtained by DP [22].

Meanwhile, PHEVs are equipped with large-capacity batteries, so efficient battery thermal management can extend battery lifetime and ensure battery safety. Optimizing thermal management strategies (TMS) is also an important method to reduce vehicle energy consumption [23]. Wei et al. found that implementing integrated energy and thermal management strategy (IETMS) for hybrid systems can improve driving economy [24]. Amini et al. established a control-oriented air conditioning (AC) system model. They used a hierarchical model predictive control to achieve IETMS, which improved the fuel economy by 2.2–5.3% under different driving conditions [25]. Fabio et al. consider the battery degradation cost in the optimization objective function of IETMS and constructs Pareto curves through convex programming collaborative optimization system matching and real-time control to balance battery degradation and energy consumption, increasing battery lifetime by more than 15% [26]. The classification of IETMS is similar to that of EMS, including rule-based, instantaneous optimization-based, and global optimization-based strategies. However, existing research on IETMS mostly focuses on instantaneous optimization-based control, lacking global optimization research for this problem. This makes it difficult to quantitatively evaluate the energy-saving potential of the real-time IETMS under different driving conditions, nor to guide the development of online control strategies. 

DP algorithm is the most common solving algorithm to obtain the global optimal power distribution solution for PHEVs. Battery state-of-charge (SOC) and engine output power are usually taken as the PHEV system state variable and control variables, respectively. Xu et al. also treated the vehicle work mode as a system state variable and added a penalty function to reduce the number of vehicle work mode switches [18]. Zhou et al. unified the EMS global optimization problem into a standardized expression based on the powertrain structural characteristics of series, parallel, and series-parallel hybrid electric vehicles [27]. However, the state variables and control variables of the PHEV system need to be discretization when solving the global optimization problem by DP. With the increase of system state variables and grid point density, the computational burden of the DP algorithm increases exponentially, resulting in a ‘Curse of dimensionality’. To alleviate this issue, Claudio et al. adaptively adjusted the grid number of SOC based on the probability distribution of battery output power under a standard driving cycle to reduce computational burden [28]. Xu et al. established a hierarchical global optimization framework of ‘information layer—physical layer—energy layer—dynamic programming’ to obtain travel information at various times. They improved the optimization performance of the DP algorithm by optimizing the actual feasible region of SOC. The calculation time was reduced by more than 25% under different driving conditions [29]. The research on improving the solution performance of the EMS global optimization problem by DP has achieved good results, but its results cannot be directly used in IETMS. This is because EMS needs to consider only one continuous state variable, SOC, while IETMS includes two continuous state variables, SOC and battery temperature, which increases computational complexity exponentially. Additionally, the state equation of the PHEV system and optimization method also have significant difference.

To address the above issues in existing research, a novel global optimization algorithm is proposed in this work to solve the IETMS global programming problem for PHEV. Firstly, the PHEV state equation with SOC and battery temperature as variables is established. Secondly, an improved dynamic programming algorithm based on adaptive grid optimization (AGO–DP) is proposed, which has a shorter computation time and higher optimization accuracy than the traditional DP algorithm. Finally, the IETMS energy-saving potential of PHEV under different driving conditions is analyzed.

The remainder of this paper is organized as follows. The control-oriented model of PHEV is developed in Section 2. Section 3 illustrates the global optimization principle based on AGO–DP. The simulation results and discussion are presented in Section 4. Finally, the conclusion is summarized in Section 5.

## 2. Control-Oriented Model of PHEV

### 2.1. Vehicle Model

**Figure 1:**
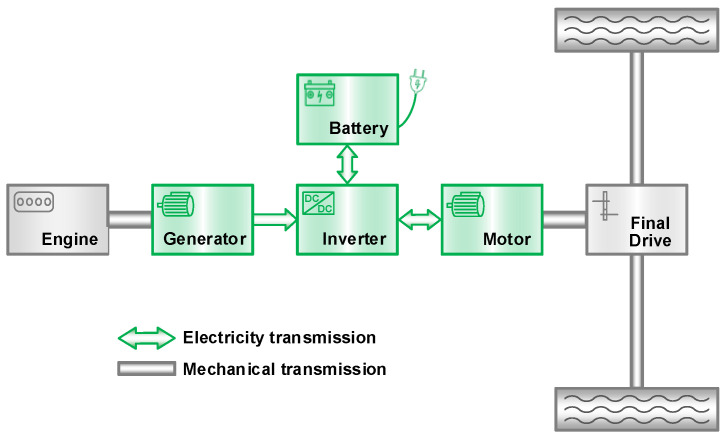
The powertrain architecture of PHEV.

The powertrain architecture of the plug-in hybrid electric sport-utility vehicle (SUV) studied in this paper is shown in Figure 1. The vehicle includes two power sources: the engine and the battery, which can provide vehicle demand power alone or together. The main parameters of the vehicle and components are shown in Table 1.

The vehicle demand power of PHEV is used to provide energy for the drive motor, AC system, and auxiliary system. Therefore, the power balance equation of PHEV can be expressed as:

(1) $$\begin{array}{c} P_{eng}+P_{bat}=P_{motor}+P_{AC}+P_{aux} \end{array}$$

where $P_{eng}$ and $P_{bat}$ denote the output power of the engine and battery. $P_{motor}$, $P_{AC}$ and $P_{aux}$ are the demand power of the drive motor, AC system, and auxiliary system, respectively. The longitudinal driving demand power accounts for the majority of the demand power during driving, so lateral demand power is ignored, and the longitudinal driving demand power of the drive motor can be calculated as follows:

(2) $$\begin{array}{c} P_{motor}=\left\{\begin{array}{c} \left(P_{f}+P_{i}+P_{w}+P_{j}\right)/\eta_{T}/\eta_{m}\;drive\;mode\\ \eta_{T}\eta_{m}\times \left(P_{f}+P_{i}+P_{w}+P_{j}\right)\;brake\;mode \end{array}\right. \end{array}$$

where $\eta_{T}$ and $\eta_{m}$ are the efficiency of the transmission system and drive motor. $P_{f}$, $P_{i}$, $P_{w}$ and $P_{j}$ are the demand power to overcome rolling resistance, slope resistance, air resistance, and acceleration resistance, which can be written as:

(3) $$\begin{array}{c} \begin{array}{c} \left\{\begin{array}{c} P_{f}=mgfcos\theta \cdot v\\ P_{i}=mgsin\theta \cdot v\\ P_{w}=\frac{1}{2}C_{D}A\rho \cdot v^{3}\\ P_{j}=\delta m\cdot a\cdot v \end{array}\right. \end{array} \end{array}$$

where m is vehicle mass, δ is the rotating mass conversion coefficient, including the translational mass and the rotating mass. $C_{D}$ is the aerodynamic drag coefficient, A is the equivalent windward area, f is rolling resistance coefficient, g is gravitational acceleration, θ is road grade, ρ is air density. v and a denote the vehicle’s instantaneous speed and acceleration, respectively.

**Figure 2:**
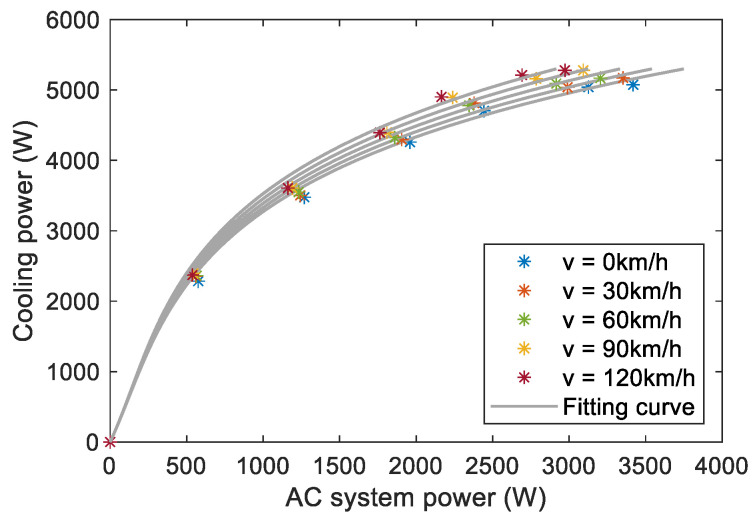
The relationship between electric power and cooling power of AC system.

Due to the complexity of the transient model of the AC system, which involves numerous state variables, it is difficult to directly apply it to global optimization. Therefore, a steady-state model is used to describe the cooling characteristics of the AC system. Specifically, if the electric power of the AC system is constant, its actual cooling power also remains constant, satisfying the following relationship:

(4) $$\begin{array}{c} P_{AC}=f_{ac}\left({\dot{Q}}_{cool}^{veh}\right)=f_{ac}\left({\dot{Q}}_{cool}^{bat}+{\dot{Q}}_{cool}^{cab}\right) \end{array}$$

where ${\dot{Q}}_{cool}^{veh}$ is the total cooling power, including cabin cooling power ${\dot{Q}}_{cool}^{cab}$ and battery cooling power ${\dot{Q}}_{cool}^{bat}$. The cooling performance of the AC system is shown in Figure 2.

**Figure 3:**
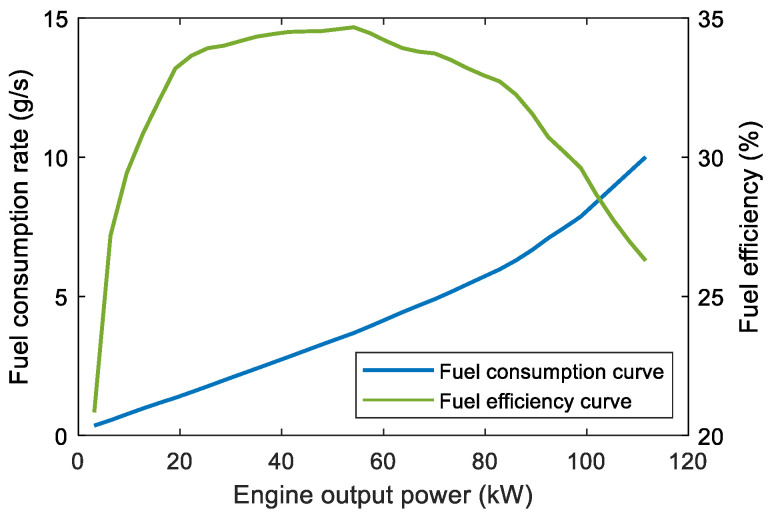
The optimal fuel consumption curve and fuel efficiency curve of the engine.

The engine working point of the PHEV series studied in this paper can be adjusted arbitrarily. Hence, the minimum fuel consumption ${\dot{m}}_{fuel}$ of the engine at different output powers can be obtained by integrating the generator efficiency, forming the optimal fuel consumption curve of the engine [30], as shown in Figure 3.

### 2.2. Battery Model

#### 2.2.1. Equivalent Circuit Model

**Figure 4:**
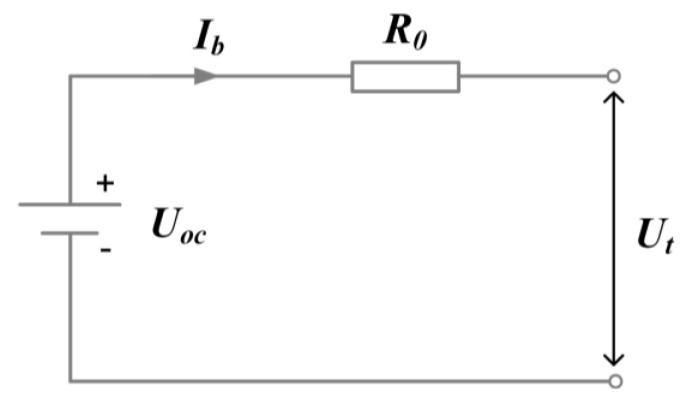
The internal resistance equivalent circuit model.

**Figure 5:**
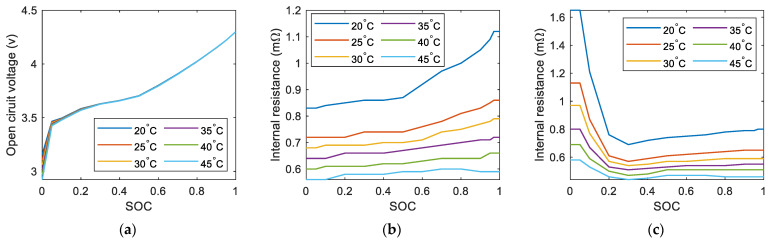
Electrical characteristic map of battery cell under different SOC and temperature: (**a**) Open-circuit voltage map; (**b**) Charging resistance map; (**c**) Discharging resistance map.

The internal resistance equivalent circuit model is used to describe the electrical characteristics of the battery, as shown in Figure 4. The expression of battery current can be calculated by Equation (5).

(5) $$\begin{array}{c} I_{b}=\frac{U_{oc}-U_{t}}{R_{0}}=\frac{U_{oc}-\sqrt{U_{oc}^{2}-4\cdot R_{0}\cdot P_{bat}}}{2\cdot R_{0}} \end{array}$$

where $U_{t}$ is the battery terminal voltage. $U_{oc}$ and $R_{0}$ are the battery’s open-circuit voltage and internal resistance. They are crucial parameters of the battery cell and can be expressed as a function of SOC and battery temperature, as shown in Figure 5.

Moreover, according to the Ampere-hour integration method, the battery SOC can be expressed as Equation (6). Where ${Cap}_{bat}$ is the battery capacity.

(6) $$\begin{array}{c} \frac{dSOC\left(t\right)}{dt}=-\frac{I_{b}\left(t\right)}{{Cap}_{bat}} \end{array}$$

#### 2.2.2. Thermal Model

The battery thermal model describes the heat generation, heat exchange, and temperature change processes of the battery [31]. According to Bernardi equation, the battery heat generation ${\dot{Q}}_{bat}$ is expressed as Equation (7). The first term in Equation (7) is the heat generation caused by the current thermal effect. The second term is reversible heat, which accounts for a small proportion of the total heat generation, so it can be ignored.

(7) $$\begin{array}{c} {\dot{Q}}_{bat}=I_{b}^{2}R_{0}+I_{b}T_{bat}\frac{\partial U_{oc}}{\partial T_{bat}} \end{array}$$

The battery heat exchange process mainly includes the heat exchange power between the battery and the external environment, as well as the heat exchange power between the battery and the AC system. After determining the heat generation power and heat exchange power of the battery, its temperature can be described as:

(8) $$\begin{array}{c} c_{p,bat}m_{bat}\frac{dT_{bat}}{dt}=I_{b}^{2}R_{0}+\frac{T_{amb}-T_{bat}}{R_{th,bat}}-{\dot{Q}}_{cool}^{bat} \end{array}$$

where $T_{amb}$ is ambient temperature. $R_{th,bat}$ = 0.0625 °C/W is the thermal resistance between the battery and the environment. $c_{p,bat}$ and $m_{bat}$ are the battery-specific heat and battery mass.

#### 2.2.3. Degradation Model

**Figure 6:**
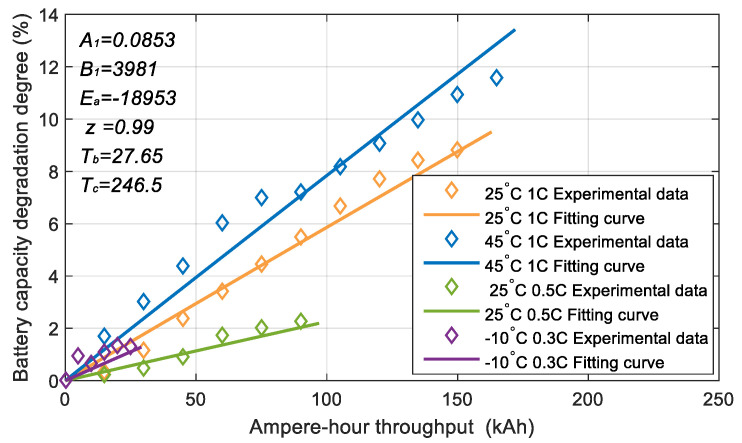
Experimental data and fitting curve of battery capacity degradation.

The empirical model based on the Arrhenius equation proposed by Song et al. [32] is used to describe the degree of battery capacity degradation ${Cap}_{loss}$, which is written as:

(9) $$\begin{array}{c} {Cap}_{loss}=A_{1}e^{-\left(\frac{E_{a}+B_{1}\cdot C_{rate}}{R\cdot \left(\left|T_{bat}-T_{b}\right|+T_{c}\right)}\right)}{\left(A_{h}\right)}^{z} \end{array}$$

where $C_{rate}=\left|I_{b}\left(t\right)\right|/Q_{bat}$ is the battery discharge rate. $A_{h}$ is the Ampere-hour throughput. R=8.31445 J/(mol × °C) is the gas constant. $E_{a}$ is the activation energy. z is the power-law factor. $T_{b}$ and $T_{c}$ are the reference temperatures. $A_{1}$ and $B_{1}$ are the fitting coefficients. For the battery cell studied in this work, four degradation cycle test conditions were set up. The battery capacity degradation curve and parameter fitting results are shown in Figure 6.

## 3. IETMS Global Optimization Based on AGO–DP 

### 3.1. Statement of IETMS Global Optimization Problem

To study the global optimization problem of IETMS, it is necessary to determine the control and state variables of the PHEV system. Considering the need to ensure the thermal comfort of the cabin during the driving process in a high-temperature environment, the cabin temperature is generally maintained in a constant suitable temperature range, which decreases significantly only in the initial driving phase. Therefore, it is assumed that the cabin temperature and cabin cooling power ${\dot{Q}}_{cool}^{cab}$ are constant during the entire driving process. The cabin cooling power ${\dot{Q}}_{cool}^{cab}$ can be calculated according to the ambient temperature and the setpoint of the AC system [33]. Furthermore, SOC, battery temperature $T_{bat}$, and engine status $S_{eng}$ are selected as the state variables x. Among them, SOC and battery temperature are continuous variables, while the engine status is a discrete variable with values of 0 or 1. Control variables u, include engine output power $P_{eng}$ and battery cooling power ${\dot{Q}}_{cool}^{bat}$, as expressed in following equation:

(10) $$\begin{array}{c} \left\{\begin{array}{c} x_{t}=\left[SOC\left(t\right),T_{bat}\left(t\right),S_{eng}\left(t\right)\right]\\ u_{t}=\left[P_{eng}\left(t\right),{\dot{Q}}_{cool}^{bat}\left(t\right)\right] \end{array}\right. \end{array}$$

The goal of PHEV energy management is to improve vehicle fuel economy. During the actual driving process of the vehicle, the battery SOC decreases and consumes electricity, leading to battery life degradation. Therefore, the total driving costs of fuel consumption $L_{fuel}$, electricity consumption $L_{ele}$, and battery degradation $L_{bat}$ are considered in the cost-to-go function instead of just considering fuel consumption which is widely applied in the previous research [5,16]. The cost-to-go function can be described as:

(11) $$\begin{array}{c} L\left(x_{t},u_{t}\right)=L_{fuel}\left(t\right)+L_{bat}\left(t\right)+L_{ele}\left(t\right)+p_{eng}\left(t\right) \end{array}$$

where $p_{eng}$ is the engine start penalty term of USD 9.55 × 10^−4^. It means that if the engine start state $S_{eng}$ changes from 0 to 1, the additional fuel consumption for engine start is considered to be 0.55 g [18] to avoid frequent engine starts and stops. Furthermore, other costs in Equation (11) are defined as follows:

(12) $$\begin{array}{c} L_{fuel}\left(t\right)=\alpha_{fuel}\times \frac{{\dot{m}}_{fuel}\left(t\right)\cdot \Delta t}{\rho_{fuel}}=\alpha_{fuel}\times \frac{b_{e,eng}\left(P_{eng}\left(t\right)\right)\cdot P_{eng}\left(t\right)\cdot \Delta t}{3600\cdot \rho_{fuel}} \end{array}$$

(13) $$\begin{array}{c} L_{bat}\left(t\right)=\alpha_{bat}\times \frac{A_{h}\left(t\right)}{A_{h,tol}\left(t\right)}=\alpha_{bat}\times \frac{\left|I_{b}\left(t\right)\right|\cdot \Delta t/3600}{A_{h,tol}\left(\left|I_{b}\left(t\right)\right|/Q_{bat},T_{bat}\left(t\right)\right)} \end{array}$$

(14) $$\begin{array}{c} \begin{array}{c} L_{ele}\left(t\right)=\alpha_{ele}\times \frac{I_{b}\left(t\right)\cdot U_{oc}\left(t\right)\cdot \Delta t}{3600\times 1000} \end{array} \end{array}$$

where $\rho_{fuel}$ = 749 g/L is fuel density. $\alpha_{fuel}$ = USD 1.3/L is the fuel cost. $\alpha_{ele}$ = USD 0.265 /kWh is the electricity price. $\alpha_{bat}$ = USD 8870.4 is the total price of the battery pack, whose unit price is set as USD 176/kWh [34].

Due to the system characteristics of vehicle components, the engine output power, battery output power, and battery cooling power are only taken within a certain range. For the state variables, their value range also needs to be limited according to the actual situation. Otherwise, the cabin thermal comfort is preferred to the battery cooling, so the maximum battery cooling power is the difference between the maximum cooling power of the AC system ${\dot{Q}}_{cool\_max}^{veh}$ and the cabin cooling power ${\dot{Q}}_{cool}^{cab}$. Then, the state equation of the PHEV system must meet the corresponding constraints:

(15) $$\begin{array}{c} \left\{\begin{array}{c} {SOC}_{min}\leq SOC\left(t\right)\leq {SOC}_{max}\\ T_{bat\_min}\leq T_{bat}\left(t\right)\leq T_{bat\_max}\\ P_{bat\_min}\leq P_{bat}\left(t\right)\leq P_{bat\_max}\\ {\dot{Q}}_{cool\_min}^{bat}\leq {\dot{Q}}_{cool}^{bat}\left(t\right)\leq {\dot{Q}}_{cool\_max}^{veh}-{\dot{Q}}_{cool}^{cab}\\ P_{eng\_min}\leq P_{eng}\left(t\right)\leq P_{eng\_max} \end{array}\right. \end{array}$$

### 3.2. Dynamic Programming Principle

**Figure 7:**
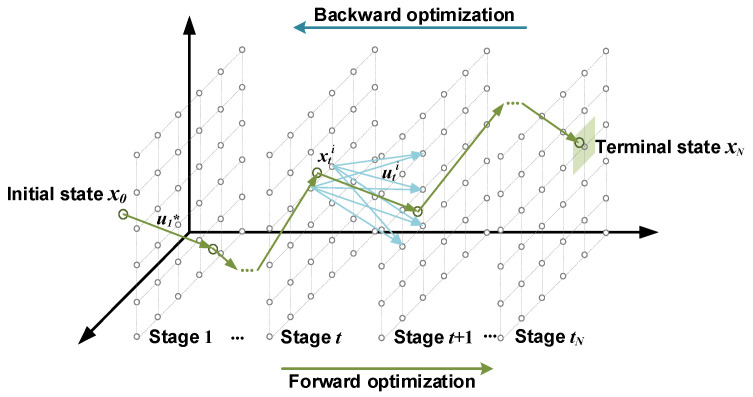
The sketch of the DP algorithm solving principle.

When solving global optimization problems using the DP algorithm, it is needed to decompose multi-stage decisions into a series of single-stage optimization problems. The initial and terminal values of state variables are determined in advance. Subsequently, to formulate the optimal decision sequence, each single-stage optimization problem is sequentially solved in a backward direction to minimize the cost-to-go function. Finally, the optimal state of each stage is calculated forward from the initial state according to the optimal decision sequence. The solving principle of the DP algorithm is shown in Figure 7. The specific solution process is as follows.

1. Calculate cost-to-go function $J^{*}(x_{t_{N}}^{i})$ of each state $x_{t_{N}}^{i}$ in the terminal state space:
  (16) $$\begin{array}{c} J^{*}\left(x_{t_{N}}^{i}\right)=J\left(x_{t_{N}}^{i}\right) \end{array}$$
2. Calculate cost-to-go function $J^{*}(x_{t}^{i})$ of each state $x_{t}^{i}$ at time t:
  (17) $$\begin{array}{c} J\left(x_{t}^{i}\right)=L\left(x_{t}^{i},u_{t}^{i}\right)+J^{*}\left(x_{t+1}^{i}\right) \end{array}$$
  where $J^{*}(x_{t+1}^{i})$ is the optimal cost-to-go function from the state $x_{t+1}^{i}$ at the t+1 time stage to the terminal state. The recursive relationship is as follows:
  (18) $$\begin{array}{c} J^{*}\left(x_{t}^{i}\right)=\underset{u_{t}\in U_{t}}{min}\left(L\left(x_{t}^{i},u_{t}\right)+J^{*}\left(x_{t+1}^{i}\right)\right) \end{array}$$
3. According to the optimal cost-to-go function $J^{*}(x_{t}^{i})$ at time t, solve the corresponding optimal control $u^{*}(x_{t+1}^{i})$:
  (19) $$\begin{array}{c} u^{*}\left(x_{t}^{i}\right)=\arg\underset{u_{t}\in U_{t}}{min}\left(L\left(x_{t}^{i},u_{t}^{i}\right)+J^{*}\left(x_{t+1}^{i}\right)\right) \end{array}$$
4. Repeat the above steps until the initial state, and complete the backward optimization process of the DP algorithm. Then, through the state equation of the PHEV system, the optimal state and optimal control sequences of each time stage are solved in a forward direction to determine the global optimal solution.

### 3.3. Adaptive Grid Optimization of State Variables

The constraints in Equation (15) specify the range of the control variables and state variables, but there is a coupling relationship between these. Due to the finite value range of the control variables, the change of the state variable SOC and battery temperature in adjacent time stages is also limited according to Equations (6) and (8), and there are no sudden changes in state variables. Therefore, if the initial and terminal values of the state variables are determined in advance, the actual feasible region of the state at each time may be less than the constraint range in Equation (15). AGO–DP is proposed in this paper to determine the actual value range of the state variables at different time stages and remove the infeasible region in the state constraints by combining the system state equation. At the same time, according to the practical, feasible region of state variables at different time stages, the interval of state grid points is adaptively adjusted to improve the accuracy of global optimization by DP without increasing the additional computation time.

**Figure 8:**
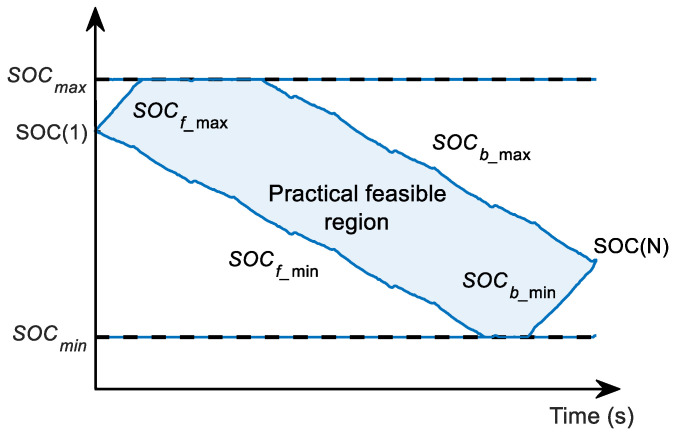
SOC practical, feasible region optimized by AGO–DP.

To reduce the range of SOC and optimize the grid distribution, it is necessary to recursively calculate the SOC variation ranges along the forward and backward directions. Furthermore, combine the upper and lower limits of SOC constraints to take the intersection of these ranges as the actual feasible region of SOC at each time stage. The specific steps are as follows:

1. The variation range of SOC at each time stage is related to the maximum and minimum output power of the battery at that time. The output power limit of the battery at time t can be calculated according to Equations (20) and (21). A positive battery output power indicates that the battery is discharging and the current is also positive; On the contrary, a negative output power indicates that the battery is charging, and the current is also negative.
  (20) $$\begin{array}{c} P_{bat\_max}\left(t\right)=P_{motor}\left(t\right)-P_{eng\_min}+f_{ac}\left({\dot{Q}}_{cool\_max}^{bat}\left(t\right)+{\dot{Q}}_{cool}^{cab}\right) \end{array}$$
  (21) $$\begin{array}{c} P_{bat\_min}\left(t\right)=P_{motor}\left(t\right)-P_{eng\_max}+f_{ac}\left({\dot{Q}}_{cool\_min}^{bat}\left(t\right)+{\dot{Q}}_{cool}^{cab}\right) \end{array}$$
2. After determining the actual limit of the battery output power, the recursive calculation in a forward direction can be performed from the SOC initial state. Then the upper and lower limits of SOC at the next time stage correspond to the minimum output power and maximum output power of the battery, which is expressed as:
  (22) $$\begin{array}{c} {SOC}_{f\_max}\left(t+1\right)=\min\left({SOC}_{f\_max}\left(t\right)-\frac{I_{P_{bat\_min}\left(t\right)}\cdot \Delta t}{Q_{bat}},\;{SOC}_{\max}\right) \end{array}$$
  (23) $$\begin{array}{c} {SOC}_{f\_min}\left(t+1\right)=\max\left({SOC}_{f\_min}\left(t\right)-\frac{I_{P_{bat\_max}\left(t\right)}\cdot \Delta t}{Q_{bat}},\;{SOC}_{\min}\right) \end{array}$$
  where $I_{P_{bat\_\max}\left(t\right)}$ and $I_{P_{bat\_\min}\left(t\right)}$ are the corresponding battery output currents when operating at maximum and minimum output power at time t.
3. The recursive calculation in a backward direction is performed from the SOC terminal state. The upper and lower limits of SOC at the previous time correspond to the maximum output power and minimum output power of the battery, respectively, which is expressed as:
  (24) $$\begin{array}{c} {SOC}_{b\_max}\left(t-1\right)=\min\left({SOC}_{b\_max}\left(t\right)+\frac{I_{P_{bat\_max}\left(t\right)}\cdot \Delta t}{Q_{bat}},\;{SOC}_{\max}\right) \end{array}$$
  (25) $$\begin{array}{c} {SOC}_{b\_min}\left(t-1\right)=\max\left({SOC}_{b\_min}\left(t\right)+\frac{I_{P_{bat\_min}\left(t\right)}\cdot \Delta t}{Q_{bat}},\;{SOC}_{\min}\right) \end{array}$$
4. According to the above method, the forward recursive boundary ${SOC}_{f\_\max/\min}$ and the backward recursive boundary ${SOC}_{b\_\max/\min}$ of SOC are obtained, respectively. The optimized limits of the SOC feasible region can be calculated by Equations (26) and (27), as shown in Figure 8.
  (26) $$\begin{array}{c} {SOC}_{opt\_max}\left(t\right)=\min\left({SOC}_{f\_max}\left(t\right),{SOC}_{b\_max}\left(t\right)\right) \end{array}$$
  (27) $$\begin{array}{c} {SOC}_{opt\_min}\left(t\right)=\max\left({SOC}_{f\_min}\left(t\right),{SOC}_{b\_min}\left(t\right)\right) \end{array}$$
5. According to the SOC feasible region range at each time stage, the SOC grid interval is adaptively adjusted to ensure that all state grid points are within the feasible region. In this paper, the state grid points are evenly distributed, and the number of SOC grid points in the global optimization problem is set to $N_{soc,grid}$. The position of SOC grid points ${SOC}_{grid}\left(t,i\right)$ can be calculated by:
  (28) $$\begin{array}{c} {SOC}_{grid}\left(t,i\right)={SOC}_{opt\_min}\left(t\right)+\frac{{SOC}_{opt\_max}\left(t\right)-{SOC}_{opt\_min}\left(t\right)}{N_{soc,grid}}\times i \end{array}$$
  $$i=0,1,2,\ldots ,N_{soc,grid}$$

When optimizing the feasible region of battery temperature during the vehicle driving process, the key lies in determining the real-time output current range, thereby calculating the maximum and minimum heat generation of the battery at each time stage. According to Equation (8), it can be seen that when the absolute value of the battery current is the highest, and there is no battery cooling power, the battery has the maximum temperature rise. Conversely, when the absolute value of the battery current is the smallest and the cooling power is the highest, the battery temperature decreases to the maximum allowable extent. The feasible region optimization steps of battery temperature are as follows:

1. If the maximum output power $P_{bat\_\max}\left(t\right)$ and minimum output power $P_{bat\_\min}\left(t\right)$ of the battery are both positive or negative at time t, the battery must be discharged or charged at this time to meet the vehicle demand power. At this time, the maximum and minimum heat generation of the battery are:
  (29) $$\begin{array}{c} {\dot{Q}}_{bat\_max}=\max\left[I_{P_{bat\_max}\left(t\right)}^{2}\cdot R_{0}\left(t\right),I_{P_{bat\_min}\left(t\right)}^{2}\cdot R_{0}\left(t\right)\right] \end{array}$$
  (30) $$\begin{array}{c} {\dot{Q}}_{bat\_min}=\min\left[I_{P_{bat\_max}\left(t\right)}^{2}\cdot R_{0}\left(t\right),I_{P_{bat\_min}\left(t\right)}^{2}\cdot R_{0}\left(t\right)\right] \end{array}$$
  If the plus–minus sign of the maximum battery output power and the minimum battery output power are opposite, it means that the battery does not need to be operated at this moment, and the demand power can be met only by the engine. Hence, the maximum heat generation can still be calculated according to Equation (29), while the minimum heat generation is 0.
2. The battery heat exchange boundary at each moment can be calculated by:
  (31) $$\begin{array}{c} P_{ex,bat\_max}\left(t+1\right)={\dot{Q}}_{bat\_max}\left(t\right)-{\dot{Q}}_{cool\_min}^{bat}\left(t\right)+\frac{T_{amb}-T_{bat,f\_max}\left(t\right)}{R_{th,bat}} \end{array}$$
  (32) $$\begin{array}{c} P_{ex,bat\_min}\left(t+1\right)={\dot{Q}}_{bat\_min}\left(t\right)-{\dot{Q}}_{cool\_max}^{bat}\left(t\right)+\frac{T_{amb}-T_{bat,f\_min}\left(t\right)}{R_{th,bat}} \end{array}$$
3. The recursive calculation in a forward direction is performed from the battery temperature’s initial state. The upper and lower boundaries of battery temperature at each time can be calculated by:
  (33) $$\begin{array}{c} T_{bat,f\_max}\left(t+1\right)=\min\left(T_{bat,f\_max}\left(t\right)+\frac{P_{ex,bat\_min}\left(t\right)\cdot \Delta t}{c_{p,bat}m_{bat}},T_{bat\_max}\right) \end{array}$$
  (34) $$\begin{array}{c} T_{bat,f\_min}\left(t+1\right)=\max\left(T_{bat,f\_min}\left(t\right)+\frac{P_{ex,bat\_max}\left(t\right)\cdot \Delta t}{c_{p,bat}m_{bat}},T_{bat\_min}\right) \end{array}$$
4. The recursive calculation in a backward direction is performed from the battery temperature terminal state. The boundaries of battery temperature can be expressed as:
  (35) $$\begin{array}{c} T_{bat,b\_max}\left(t-1\right)=\min\left(T_{bat,b\_max}\left(t\right)-\frac{P_{ex,bat\_max}\left(t\right)\cdot \Delta t}{c_{p,bat}m_{bat}},T_{bat\_max}\right) \end{array}$$
  (36) $$\begin{array}{c} T_{bat,b\_min}\left(t-1\right)=\max\left(T_{bat,b\_min}\left(t\right)-\frac{P_{ex,bat\_min}\left(t\right)\cdot \Delta t}{c_{p,bat}m_{bat}},T_{bat\_min}\right) \end{array}$$
5. According to the forward recursive boundary $T_{bat,f\_\max/\min}$ and backward recursive boundary $T_{bat,b\_\max/\min}$, the limits of the battery temperature feasible region can be calculated by:
  (37) $$\begin{array}{c} T_{bat,opt\_max}\left(t\right)=\min\left(T_{bat,f\_max}\left(t\right),{\;T}_{bat,b\_max}\left(t\right)\right) \end{array}$$
  (38) $$\begin{array}{c} T_{bat,opt\_min}\left(t\right)=\max\left(T_{bat,f\_min}\left(t\right),{\;T}_{bat,b\_min}\left(t\right)\right) \end{array}$$
6. Similarly, the state grid points of battery temperature are evenly distributed. The number of grid points of battery temperature is set to $N_{Tbat,grid}$, and the position of grid points $T_{bat,grid}\left(t,i\right)$ is:
  (39) $$\begin{array}{c} T_{bat,grid}\left(t,i\right)=T_{bat,opt\_min}\left(t\right)+\frac{T_{bat,opt\_max}\left(t\right)-T_{bat,opt\_min}\left(t\right)}{N_{Tbat,grid}}\times i \end{array}$$
  $$i=0,1,2,\ldots ,N_{Tbat,grid}$$

## 4. Simulation Results and Discussion

### 4.1. Comparison of Global Optimization Algorithms

**Figure 9:**
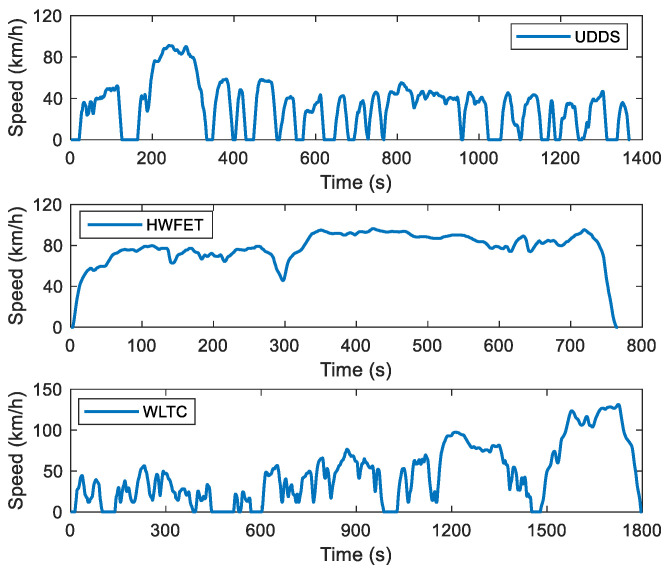
Speed curve of test cycle in simulation analysis.

In order to verify the superiority of AGO–DP in solving the IETMS global optimization problem of PHEV, three standard driving cycles are selected as test cycles: urban dynamometer driving schedule (UDDS), highway fuel economy test driving cycle (HWFET) and world light-duty vehicle test cycle (WLTC), and the speed curves are shown in Figure 9. These test cycles include high-speed driving conditions, low-speed driving conditions, and a combination of high and low-speed driving conditions, which can better cover various scenarios in daily driving.

The simulation time step is set to 1 s to discretize the driving condition. The ambient temperature and the light intensity are set to 35 °C and 800 W/m^2^, and the setpoint temperature of the cabin is 25 °C. According to the cabin thermal load expression proposed by Zhao [33], the cabin cooling power is 2320 W at this condition. The initial battery temperature is consistent with the ambient temperature, and the battery terminal temperature should be at an appropriate temperature to rapidly obtain a large current when fast charging after driving, so it is set to 25 °C [35]. The SOC at the end of the driving condition should be kept at a low level to fully consume electricity and improve the vehicle energy efficiency, so the initial and terminal SOCs are set to 0.85 and 0.45, respectively. For the IETMS global optimization problem of PHEV, the relevant parameters of state and control variables are listed in Table 2. All computations in this work are performed on an Intel Core i7-11800H CPU at 2.30 GHz with 16 GB RAM and 64 bits system.

**Figure 10:**
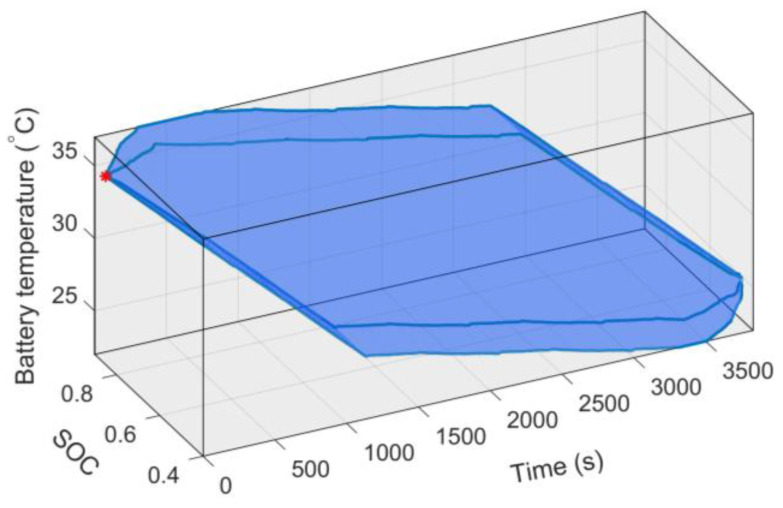
Practical, feasible region of state variables optimized by AGO–DP.

The difference between the AGO–DP algorithm and the traditional DP algorithm is mainly reflected in the feasible region of the state variables. Taking the simulation result of the 5 × HWFET driving condition as an example, the practical, feasible region of SOC and battery temperature during the entire driving process optimized by AGO–DP is shown in Figure 10. The reduction effect of the state feasible range is most significant during the initial and final stages of the driving condition, and its range is much smaller than the limits of state variables in Table 2. This is because the initial and final values of system state variables are determined, and the driving demand power, engine output power, and battery cooling power all have limited value ranges. Therefore, the change rates of SOC and battery temperature are finite without sudden change, and their feasible regions gradually expand until reaching the state variable limits.

The three test cycles in Figure 9 are repeated several times. Meanwhile, AGO–DP is used for global optimization, and the reduction ratio of state feasible region can be calculated as follows:

(40) $$\begin{array}{c} \eta_{range}=1-\frac{\sum_{t=1}^{t_{N}}\left({SOC}_{opt\_max}-{SOC}_{opt\_min}\right)\left(T_{bat,opt\_max}-T_{bat,opt\_min}\right)}{\sum_{t=1}^{t_{N}}\left({SOC}_{max}-{SOC}_{min}\right)\left(T_{bat\_max}-T_{bat\_min}\right)} \end{array}$$

**Figure 11:**
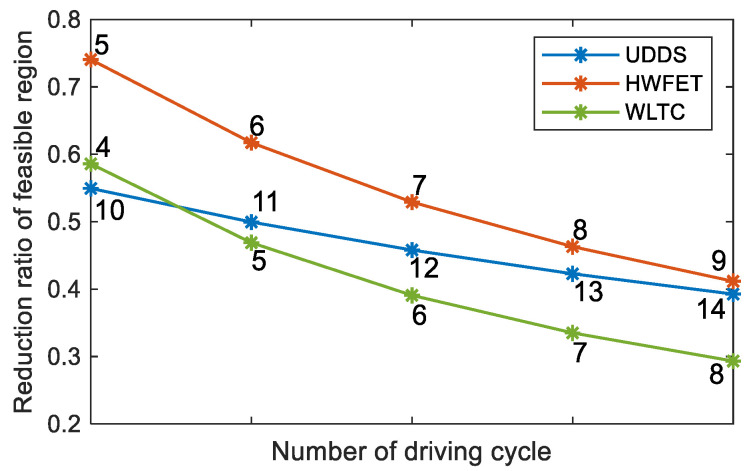
Reduction ratio of feasible region under various driving conditions.

It can be seen from Figure 11 that AGO–DP can effectively reduce the feasible region of state variables under various driving conditions, but the reduction ratio varies among different types of driving conditions. The reduction ratio of HWFET is particularly significant, with a maximum reduction ratio of over 70%. This is because when the energy consumption of the entire driving process is at the same level, the high-speed driving condition requires higher driving power and lasts for a shorter time. It results in a shorter time range between the upper and lower limits of the state variables and a higher proportion of feasible region reduction. As the driving cycle number increases, the duration of the state feasible region boundary consistent with the state limit is longer, so the effect of feasible region reduction is weakened. However, AGO–DP can still maintain a reduction ratio of nearly 30%.

**Figure 12:**
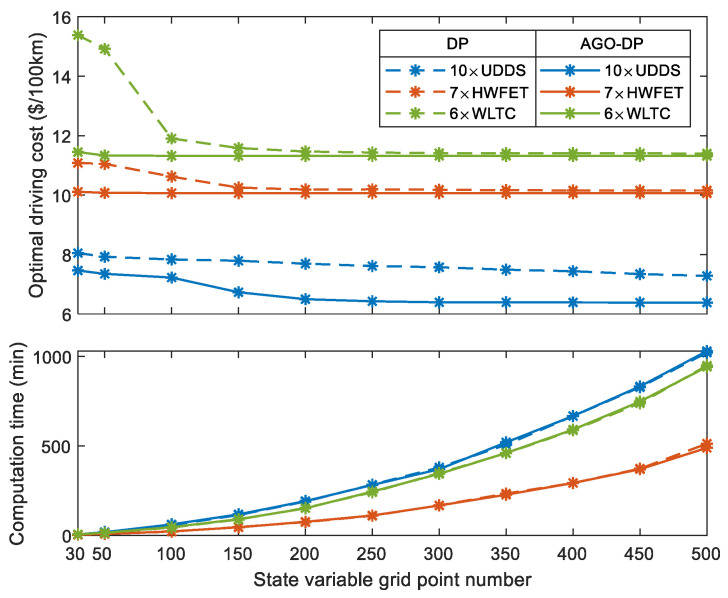
The impact of state grid point number on the optimal driving cost and computation time.

To verify the impact of state-feasible region reduction on global optimization performance, three sets of driving conditions are selected for simulation analysis under different grid point numbers of the state SOC and battery temperature variables. The traditional DP and AGO–DP are used for IETMS global optimization, respectively. The global optimal solution and computation time vary with the state grid point numbers, as shown in Figure 12. The global optimal solution is the optimal driving cost under the corresponding driving condition, and to eliminate the impact of driving distance on the optimal solution, it is normalized to driving cost per 100 km.

The global optimization performance of AGO–DP is significantly better than that of the traditional DP algorithm under various driving conditions. The optimal driving cost of AGO–DP decreases slightly with the increase of the grid point number, and its convergence rate is faster. For the driving conditions of WLTC and HWFET, even if the state variable grid is only 30, an ideal optimal cost can also be obtained. The optimal cost is basically stable when the grid point number reaches 50. However, DP has poor optimization performance when the grid point number is less than 100, and its convergence rate of the optimal cost is also slow. Meanwhile, the optimal driving cost of DP in a stable stage is slightly higher than AGO–DP. This is because the state grid points of DP are uniformly distributed within the limits of state variables, resulting in a large number of state grid points of DP being outside the actual feasible region, especially in the initial and final stages of the driving conditions. Not only does it increase the unnecessary computational burden, but it also has varying degrees of impact on the global optimization accuracy of each driving condition.

**Figure 13:**
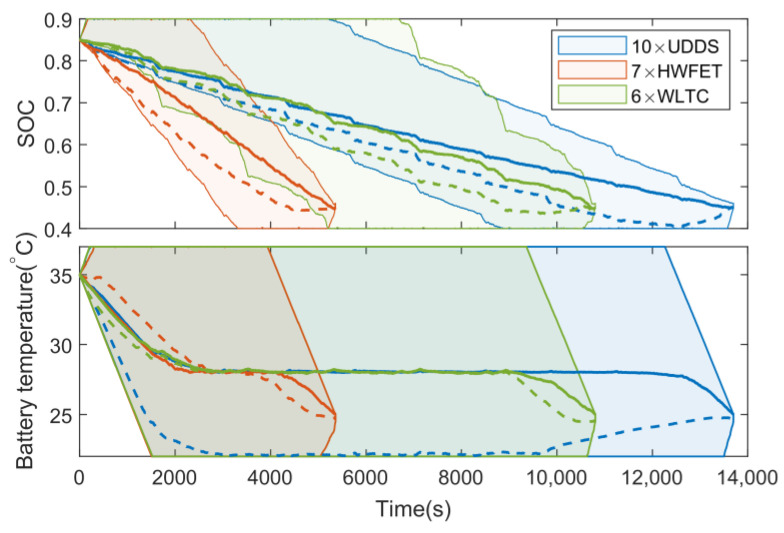
The practical, feasible regions and optimal trajectories of state variables under various driving conditions.

The two algorithms have the greatest difference in global optimization performance for UDDS. Due to the low driving demand power of UDDS, the descent of SOC feasible region boundary is the slowest, as shown in Figure 13, which minimizes the state grid point number in the feasible region for global optimization. Meanwhile, UDDS has the longest driving time, which causes greater interpolation cumulative errors and reduces optimization accuracy. AGO–DP adaptively adjusts the distribution of the state grid points according to the actual feasible region at different times, ensuring that all grid points are within the feasible region. It also refines the grid interval of the feasible region, thereby reducing interpolation errors and making the cost-to-go function of backward optimization more accurate. Therefore, AGO–DP can obtain the global optimal solution more rapidly and accurately. The driving demand power of HWFET and WLTC is larger than that of UDDS, so the boundary of the SOC feasible region decreases rapidly. It makes the difference between the optimized feasible region and the limits of state variables not significant during the initial and final stages of the driving condition. The traditional DP algorithm also has a large number of state grid points falling in the feasible region. Hence, its interpolation error is smaller than that of UDDS, and the optimal driving cost is close to AGO–DP. However, the convergence rate of the optimal solution is still significantly slower than that of AGO–DP.

**Figure 14:**
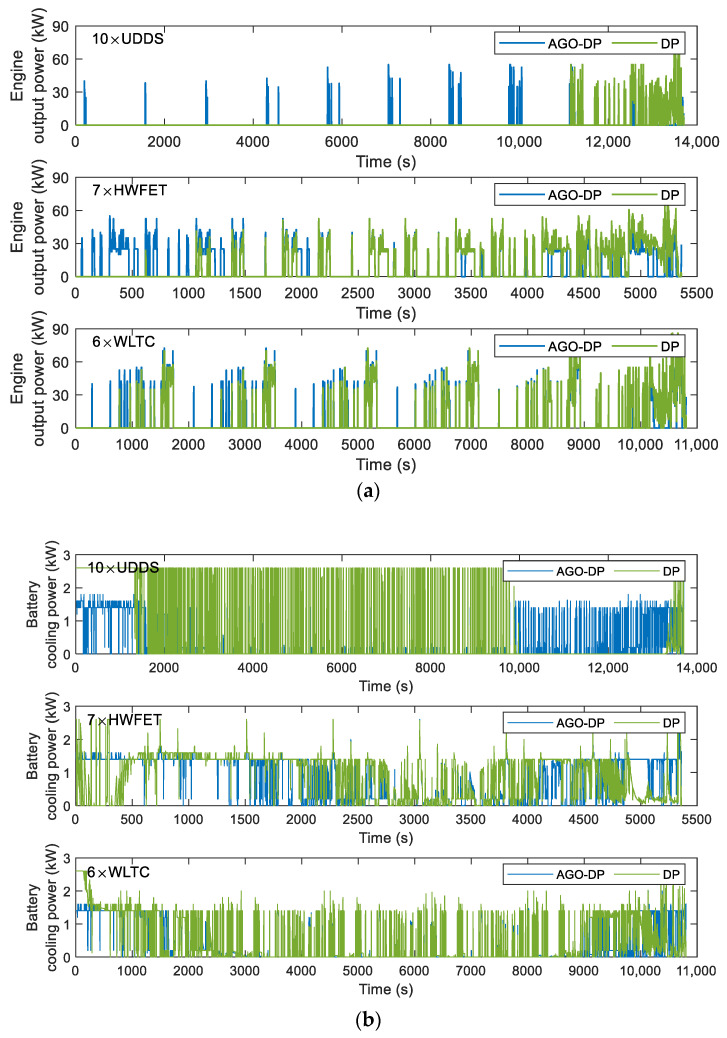
Optimal trajectories of the control variables under various driving conditions: (**a**) engine output power and (**b**) battery cooling power.

The solid and dotted lines in Figure 13 are the optimal state trajectories obtained by AGO–DP and DP with the state grid points of 250, respectively. Figure 14 shows the optimal trajectories of engine output power and battery cooling power. The decline of SOC optimal trajectory of DP is faster than that of AGO–DP, which indicates that the battery outputs most of the vehicle demand power in the early stage of the driving condition, and the engine is less involved. The overall driving condition characteristics are not well considered by DP. The battery temperature trajectory obtained by DP under UDDS differs significantly from AGO–DP. It is also due to the smaller number of state grid points in the feasible region during the initial and final stages, resulting in significant interpolation errors. The global optimization performance difference exceeds more than 10% compared to AGO–DP. Furthermore, most of the engine output power of AGO–DP is between 30–60 kW, which is a high-efficient engine range, according to Figure 3. However, in the optimal control trajectory of DP, the engine often operates with a power above 60 kW during the final stage, decreasing fuel efficiency. Otherwise, the efficiency of the AC system decreases with an increase in cooling power, according to Figure 2, so the battery cooling power is usually maintained at around 1.5 kW to reduce energy consumption. The battery cooling power operates at maximum power only in the DP optimization result under UDDS driving conditions, resulting in much worse economy.

It can also be seen from Figure 12 that the calculation time of global optimization shows an exponential growth trend. When the state grid point number is the same, the calculation time of the two algorithms is not significantly different because AGO–DP only adjusts the grid point distribution within the practical state feasible region but does not reduce the number of grid points. The calculation amount of the two algorithms is basically the same, so the calculation time is also close. However, AGO–DP only requires fewer grid points to achieve a stable optimal driving cost than DP. Under the three types of driving conditions, AGO–DP can obtain the optimal cost with the grid point numbers of 200, 50, and 50, respectively. However, DP needs 250, 150, and 150, and the time-saving ratio of AGO–DP compared to DP is 33.29%, 83.33%, and 84.67%, respectively. Meanwhile, the optimal driving cost solved by the two algorithms under different driving conditions is shown in Table 3, and the state grid point number is set to 150.

The IETMS global optimization performance of AGO–DP is significantly better than that of DP, and the average difference between the two algorithms is 6.99%. UDDS has the largest reduction ratio of feasible regions, resulting in the largest reduction of 16.85% in driving costs. In other types of driving conditions, the advantage of AGO–DP has weakened, but it is still better than the traditional DP algorithm.

### 4.2. IETMS Energy-Saving Potential Analysis

**Figure 15:**
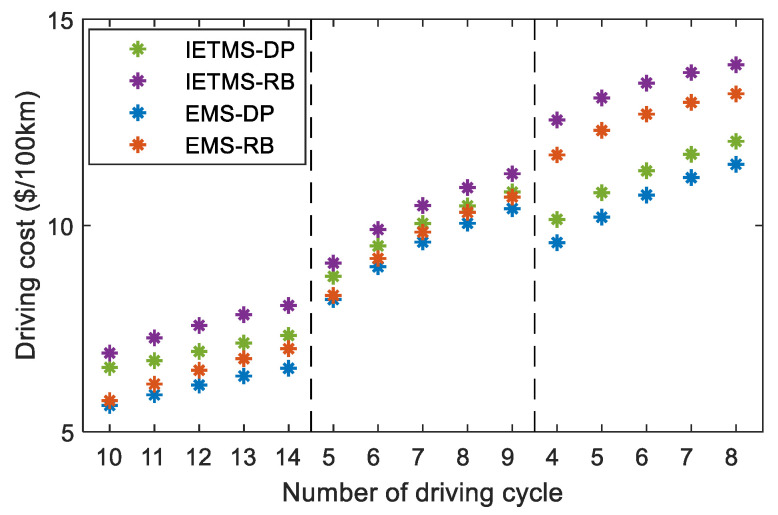
Comparison of driving costs under different strategies.

To illustrate the importance of developing IETMS for PHEVs, the energy-saving potential of IETMS and EMS global optimization is compared in this section. IETMS-DP strategy conforms to the development of the IETMS global optimization mentioned above. EMS-DP strategy only does EMS global optimization and ignores battery thermal management. Meanwhile, rule-based EMS (EMS-RB) and rule-based IETMS (IETMS-RB) are introduced for comparative analysis. Rule-based strategy uses charge-depleting and charge-sustaining (CD/CS) control [36] and proportion-integral-differential (PID) control to achieve power distribution and battery thermal management for PHEVs, respectively. In the CD stage, the battery is regarded as the only power source unless the driving demand power exceeds the maximum battery output power. The goal of the CS stage is to stabilize the battery SOC at 0.45. The engine output power can be adjusted according to SOC, expressed in Equation (41). Figure 15 shows the driving cost under various driving conditions obtained by different strategies.

(41) $$\begin{array}{c} P_{eng}=P_{cs,chg}\times \left(\frac{SOC_{cs,hi}+SOC_{cs,lo}}{2}-SOC\right) \end{array}$$

where $P_{cs,chg}$ = 50 kW is the desired output power during engine operation. $SOC_{cs,hi}$ and $SOC_{cs,lo}$ are the SOC threshold for correcting engine power, set to 0.6 and 0.3, respectively. 

**Figure 16:**
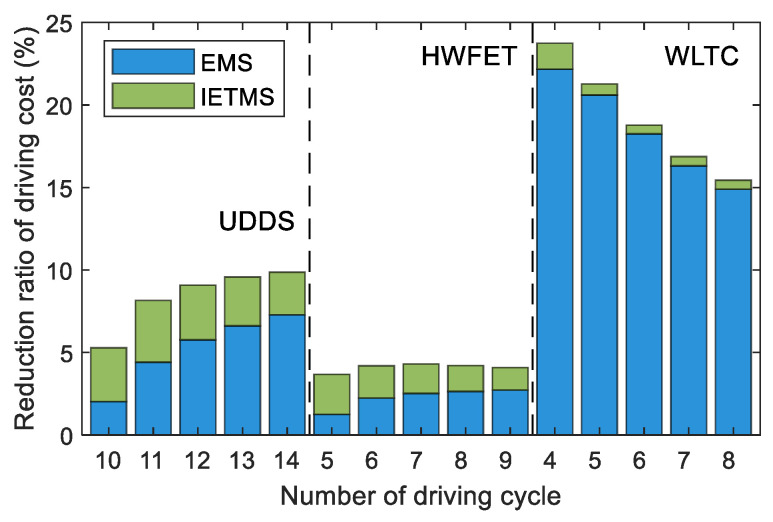
Energy-saving potential of IETMS and EMS under various driving conditions.

The driving cost increases linearly with the number of driving cycles. This is because the energy the battery provides is the same in different driving conditions. As the number of cycles increases, the proportion of engine energy supply gradually increases, leading to a gradual increase in fuel consumption and driving cost. Although battery thermal management improves battery efficiency and slows down battery aging, the energy consumption cost of battery thermal management is higher than the cost of reducing battery aging. Therefore, the cost of IETMS is higher than that of EMS under the same driving condition. However, battery thermal management is crucial for the long-term health and safety of batteries, so although it increases the driving cost, it is indispensable. Figure 16 shows the proportion of cost reduction based on the global optimization strategy compared to the rule-based strategy, which can measure the energy-saving potential of different strategies. The larger the difference in driving costs between the rule-based strategy and the global optimization strategy, the greater the energy-saving potential. The decrease in the proportion of battery energy supply leads to a gradual decrease in battery heat generation. The demand for battery thermal management is reduced. Therefore, the energy-saving potential of IETMS compared to EMS gradually decreases.

The distribution of driving costs varies under different driving conditions. The high-speed driving demand power of HWFET is relatively high, and the engine can operate in a high-efficient region in various strategies. The energy consumption for the battery thermal management is relatively small compared to the driving energy consumption, so the driving cost difference of various strategies is the smallest, and the energy-saving potential is also the smallest. Under this driving condition, the average energy-saving potential of EMS is only 2.28%, while the energy-saving potential of IETMS can reach over 4%. This is because, considering the thermal management, the battery can be cooled down to the appropriate temperature range, thereby reducing the battery capacity degradation caused by the high current and high-temperature operation, reducing driving costs, and improving energy-saving potential. The urban driving condition of UDDS is mainly low speed, with low driving demand power. The battery is the main power source, so the driving cost is the lowest. Moreover, the thermal management energy consumption under this condition accounts for a relatively large proportion, so the average energy-saving potential of IETMS has increased to 8.39%, which is 3.17% higher than EMS. The comprehensive driving conditions of WLTC include different speed ranges, making it difficult to achieve a good driving economy because of significant fluctuations in engine output power based on rule-based strategies. Global optimization can balance the output power of the engine and power battery based on global driving conditions to achieve optimal driving costs. This driving condition has the most obvious energy-saving potential, reaching a maximum of over 20%. Meanwhile, IETMS only improves by 0.55–1.57% compared to EMS.

## 5. Conclusions

An improved DP algorithm is proposed in this paper to solve IETMS global optimization problem for PHEVs by reducing the optimization range of SOC and battery temperature and adaptively adjusting the grid distribution of state variables according to the actual feasible region. The feasible region of state variables under different driving conditions is effectively optimized by AGO–DP. The maximum reduction ratio of the feasible region reaches 74.1% of HWFET. Although the effect of feasible region reduction is weakened as the increase of driving cycle number, the reduction ratio is still nearly 30%, which reduces the computational burden for IETMS global optimization. Meanwhile, AGO–DP can obtain better global optimal driving costs more rapidly and accurately than DP. The global optimization computation time is reduced by 33.29–84.67%, and the accuracy of the global optimal solution is improved by 0.94–16.85%. The engine and AC system in AGO–DP both operate in a more efficient region. Furthermore, the IETMS energy-saving potential range for PHEVs is 3.68–23.74% under different driving conditions, which increases the energy-saving potential by 0.55–3.26% compared to EMS. In future research, AGO–DP proposed in this work will be used as a reference benchmark to guide the development of an online control strategy and further improve vehicle energy efficiency.

## Figures and Tables

**Table 1:** The main parameters of the vehicle and components.

Component	Parameter	Value	Unit
Vehicle	Curb weight	3120	kg
	Rotating mass conversion coefficient	1.1	-
	Aerodynamic drag coefficient	0.6	-
	Equivalent windward area	3.25	m^2^
	Rolling resistance coefficient	0.009	-
	Transmission efficiency	0.95	-
	Final drive ratio	16.32	-
Engine	Max power/speed	130/5200	kW
	Max torque/speed	300/3500	Nm
Generator	Max power	165	kW
	Max speed	13,000	rpm
	Max torque	350	Nm
Drive Motor	Max power	195	kW
	Max speed	14,000	rpm
	Max torque	350	Nm
Battery Pack	Nominal capacity	155	Ah
	Nominal energy	54.3	kWh
	Nominal voltage	350.4	V
	Specific heat	1100	J/(kg × °C)
	Mass	267	kg

**Table 2:** The parameters of state and control variables in IETMS global optimization.

Parameter	Value
Initial SOC	0.85
Terminal SOC	0.45
Limits of SOC	[0.4:0.9]
Initial battery temperature	35
Terminal battery temperature	25
Limits of battery temperature	[22:37]
Grid points of engine output power	[0:1000:11,000]
Grid points of battery cooling power	[0:200:2600]

**Table 3:** Comparison of optimal driving cost under different driving conditions.

Driving Condition	Cycle Number	Optimal Driving Cost ($/100 km)		
		DP	AGO–DP	
UDDS	10	7.89	6.56	−16.85%
	11	8.06	6.72	−16.53%
	12	8.27	6.95	−15.97%
	13	8.16	7.15	−12.32%
	14	8.10	7.34	−9.46%
HWFET	5	9.42	8.76	−6.95%
	6	10.05	9.50	−5.42%
	7	10.29	10.05	−2.30%
	8	10.60	10.48	−1.17%
	9	10.91	10.81	−0.94%
WLTC	4	10.80	10.15	−6.07%
	5	11.44	10.80	−5.59%
	6	11.59	11.33	−2.30%
	7	11.92	11.73	−1.60%
	8	12.20	12.04	−1.31%

## Data Availability

Not applicable.

## Abbreviations

The following abbreviations are used in this manuscript:

AC	Air conditioning
AGO–DP	Dynamic programming based on adaptive grid optimization
CD/CS	Charge depleting and charge sustaining
DP	Dynamic programming
EMS	Energy management strategy
HWFET	Highway fuel economy test driving cycle
IETMS	Integrated energy and thermal management strategy
PHEV	Plug-in hybrid electric vehicle
PID	Proportion integral differential
SOC	State of charge
SUV	Sport-utility vehicle
TMS	Thermal management strategy
UDDS	Urban dynamometer driving schedule
WLTC	World light-duty vehicle test cycle
